# The influence of a weight-bearing platform on the mechanical behavior of two Ilizarov ring fixators: tensioned wires vs. half-pins

**DOI:** 10.1186/1749-799X-6-61

**Published:** 2011-12-12

**Authors:** Jan Gessmann, Mustafa Citak, Birger Jettkant, Thomas A Schildhauer, Dominik Seybold

**Affiliations:** 1 BG-University Hospital Bergmannsheil, Department of General and Trauma Surgery, Bürkle-de-la-Camp-Platz 1, 44789 Bochum, Germany; 2 BG-University Hospital Bergmannsheil, Department of Surgical Research, Bürkle-de-la-Camp-Platz 1, 44789 Bochum, Germany

## Abstract

**Background:**

A weight-bearing platform applied at the distal end of an Ilizarov external frame allows patients with hindfoot transfixations, foot deformities or plantar skin lesions to bear weight. This leads to an indirect loading of the fracture or osteotomy site. However, the effect on the fracture/osteotomy site's motion or compressive loads is unknown. The aim of this study was to analyze the mechanical effects of a weight-bearing platform on the traditional all-wire, four-ring frame in comparison to a two-ring frame consisting of half-pins.

**Methods:**

Two frame configurations, with either anatomically positioned wires or half-pins, were analyzed with and without a weight-bearing platform applied underneath the distal ring. Composite tibiae with a mid-diaphyseal osteotomy of 3.5 mm were used in all the experiments. An axial load was applied with the use of a universal test machine (UTS^®^). Interfragmentary movements, the relative movements of bone fragments and movements between rings were recorded using displacement transducers. Compressive loads at the osteotomy site were recorded with loading cells.

**Results:**

Indirect loading with a weight-bearing platform altered the force transmission through the osteotomy. Indirect loading of the tibiae decreased the extent of the axial micro-motion by 50% under the applied weight load when compared to direct weight loading (p < 0.05). The half pin frame was 25% stiffer than the wire frame under both direct and indirect loading of the tibiae (p < 0.05). Compressive loads under indirect loading were reduced by 67% in the wire frame and by 57% in the half-pin frames compared to direct loading of the bones (p < 0.05). While axial loading in the wire frames resulted in plain axial movements at the site of the osteotomy, it was coupled with translational movements and angular displacements in the half pin mountings. This effect was more apparent in the case of indirect loading.

**Conclusions:**

A weight-bearing platform has substantial influence on the biomechanical performance of an Ilizarov external fixator. Half-pins induce greater stiffness to the Ilizarov external fixator and allow the usage of only one ring per bone segment, but shear stresses at the osteotomy under axial loading should be considered. The results allow an estimation of the size and direction of interfragmentary movements based on the extent of weight bearing.

## Background

**Figure 1:**
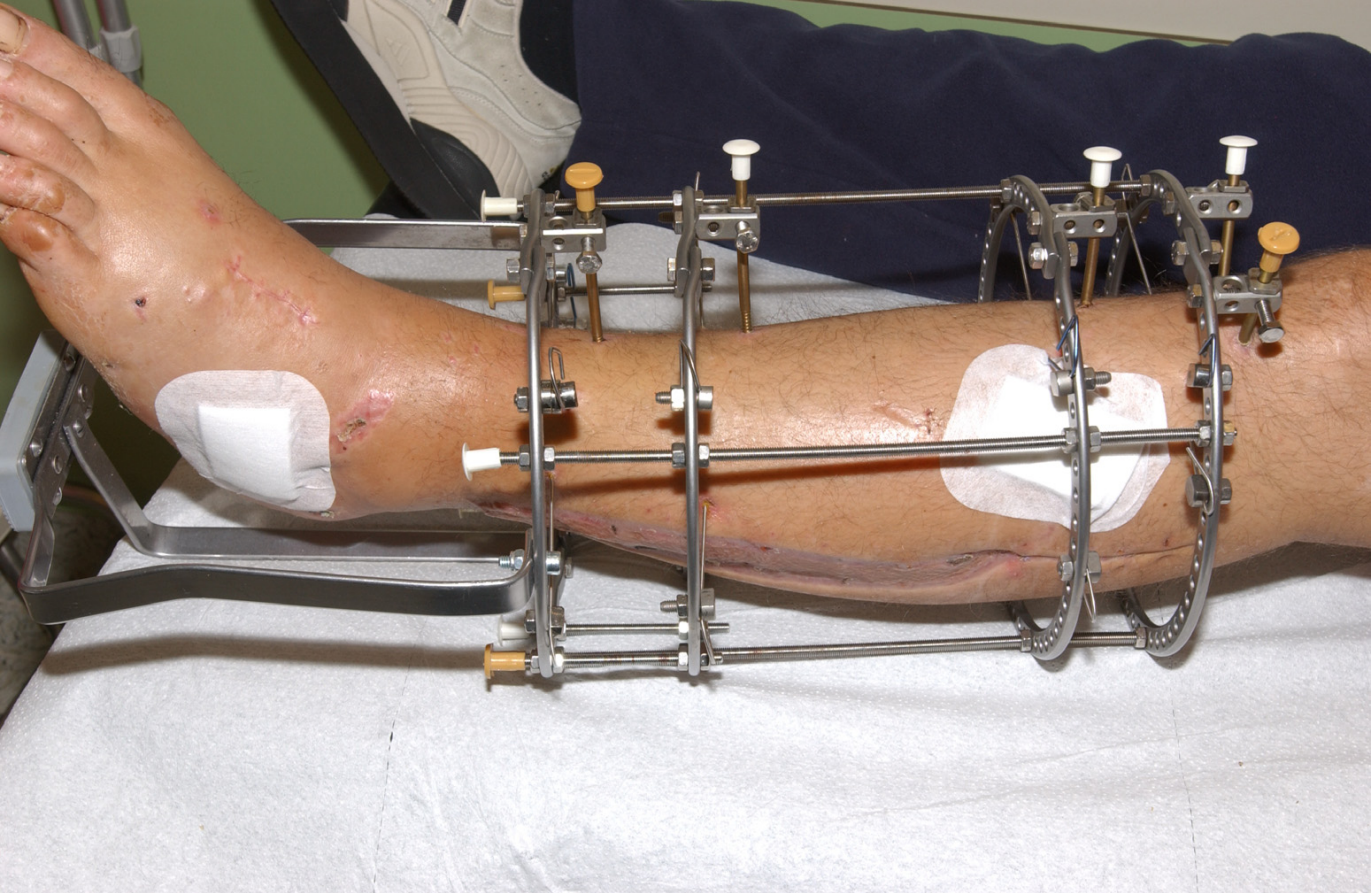
**Clinical picture of a patient treated for mid-diaphyseal fractures with a weight-bearing platform because of foot ulcera and an insensate foot sole after compartment syndrome**.

The rate and pattern of fracture healing is influenced by the nature of axial loading [1]. Weight bearing with an Ilizarov external fixator results in axial compressive loading and micro-movements at the fracture/osteotomy site [2,3] that have a beneficial effect on bone healing [4]. However, not every patient with an Ilizarov frame is able to walk with the frame due to its bulky construction, the transfixion of soft tissue by the tensioned wires or the transfixion of the hindfoot within the frame. Efforts have been made to simplify both the application and configuration of the frames to improve patient comfort while retaining an appropriate combination of stability and dynamics within the system [5]. Reducing the number of wires decreases infection rates and soft tissue impalement but simultaneously decreases frame stability [6]. Half-pins have been adapted to the Ilizarov frame because their biomechanical properties allow for the creation of a more rigid frame and a reduction in soft tissue complications [5-9]. The half-pins are used in combination with wires or as pin-only mountings [5,6]. The so-called Rancho technique allows the half-pins to be offset with respect to the ring level using blocks [9]. This simplifies frame application by the use of three pins on one ring for each bone segment rather than four wires and two rings [5,10]. Many investigators are currently examining alternative pin placement strategies that may allow for the use of fewer half-pin connections [5,11,12]. Another possibility that may improve patient comfort or even make weight bearing possible for the patient is the application of a weight-bearing platform to the distal end of the frame in the case of a transfixed foot or soft tissue complications (Figure 1) [13,14]. Due to the modularity and variability of various frame mountings, which allow the orthopedic surgeon to adapt to any clinical situation, various frame configurations are in clinical use and exhibit good healing rates, but complications such as non- and mal-union continue to occur [15-18]. One cause of failure that may be detrimental to healing is the presence of excessive shear stresses produced by asymmetrical axial fracture site motion [19,20]. On the other side, excessive rigidity may delay or even inhibit bone healing [21,22].

The aim of this study was to compare the axial stiffness of a two-ring, half-pin frame with that of the traditional four-ring, all-wire frame. Additionally, the biomechanical effects of a weight-bearing platform on the interfragmentary movements and compressive loads in the osteotomy were analyzed for each style of frame mounting.

## Methods

Composite tibiae (3^rd ^generation Sawbones^®^) were used for all the experiments. The composite bones were stabilized by two different frame mountings: a standard four-ring frame with two 1.8-mm wires (Smith and Nephew^®^) per ring and a two-ring frame with three 6-mm half-pins (Orthofix^®^) per ring. The diameter of all rings (Smith and Nephew^®^) was 160 mm, and they were connected via four threaded rods equidistant from one another.

The positioning of the wires and half-pins was performed with respect to the anatomical conditions, and the tibiae were mounted eccentrically in the sagittal plane to mimic the soft tissues of the calf. At the site of the osteotomy, the distance between the bone and the ring measured 4.5 cm from anterior in the sagittal plane. In the frontal plane, the bones were centered.

**Figure 2:**
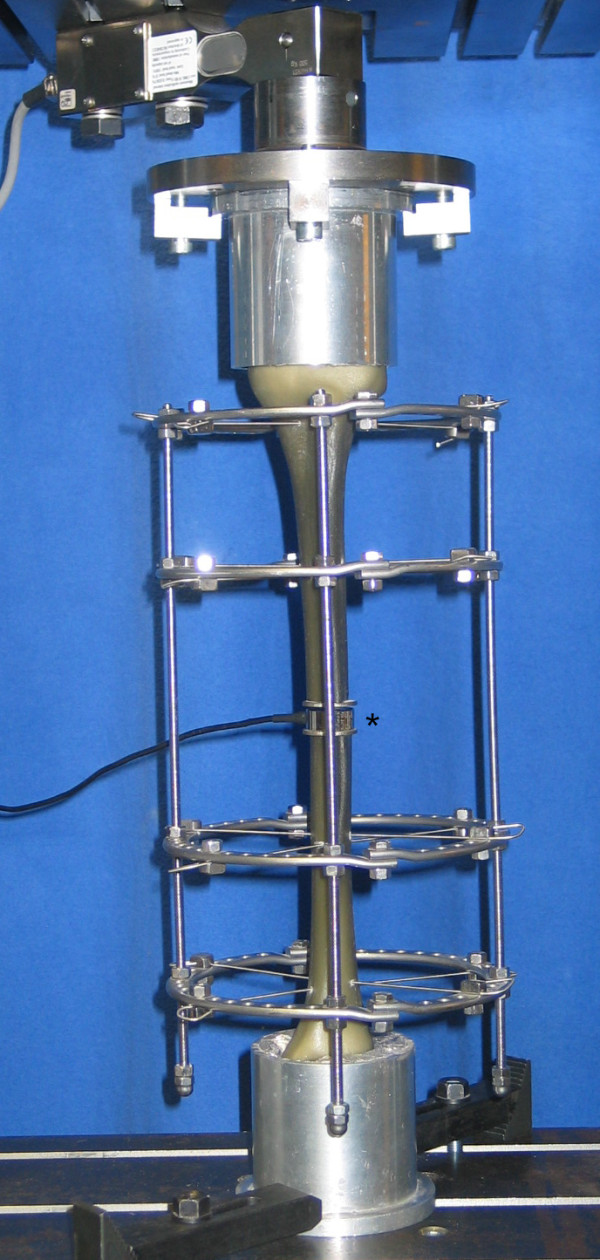
**Experimental set-up of the wire frame under direct loading (both bone ends are mounted to the test machine) for measurements of the compressive loads in the osteotomy; the loading cell is placed into the osteotomy gap**. (*) marks the loading cell in the osteotomy gap.

Wires were drilled to have a 60° angle crossing in the center of the bone, with one wire on the top side and one on the bottom side of the ring. They were attached to the ring with slotted bolts and tensioned to 1080 Nm with the standard tensioning device that is part of the Ilizarov set. The slotted bolts were tightened with a torque of 10 Nm. The distance between the two rings of each bone segment measured 6 cm (Figure 2).

**Figure 3:**
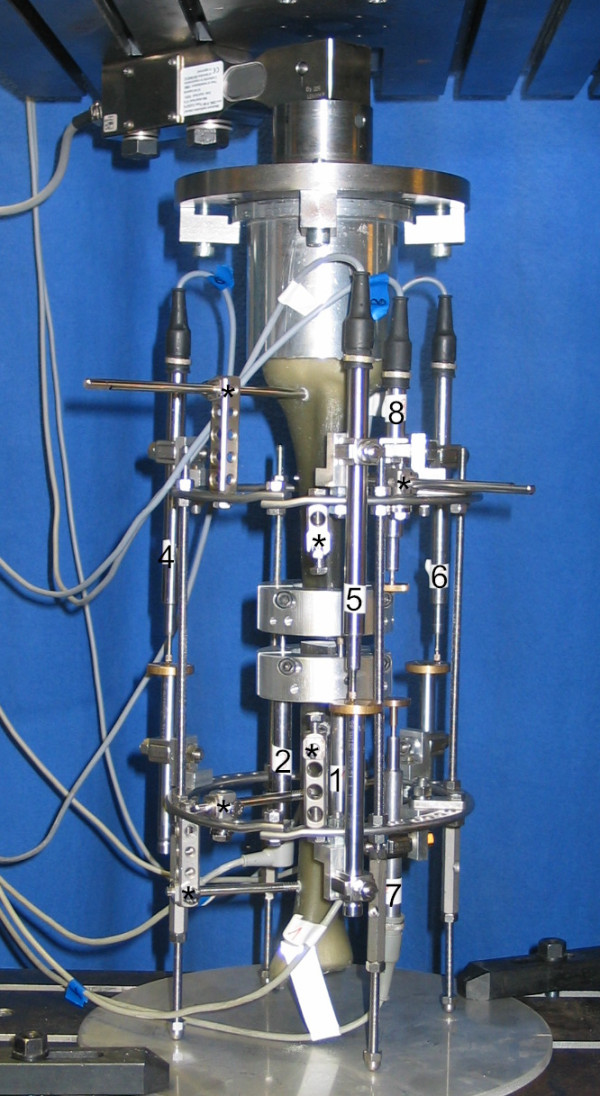
**Experimental setup of the half-pin frame under indirect loading with displacement transducers**. The connecting rods are distally extended to leave the distal bone fragment without direct contact to the base plate. The numbers label the arrangement of displacement transducers: 1-3: interfragmentary movements in the osteotomy (displacement transducer 3 out of sight behind the composite bone); 4-6: relative movement between rings; 7-8: relative movements of bone segments in relation to the ring level; (*) marks the Rancho cubes with half-pins.

The half-pin mounting utilized three pins on each of the two rings. After predrilling the composite bones, half-pins were inserted bicortically through the anterior medial cortex. The proximal and distal half-pin of each bone segment were drilled at a 90° angle to the coronal plane, with the third pin bisecting the first two pins. They were attached to the rings using Rancho cubes (Smith and Nephew^®^) that allowed the pins to be perpendicularly offset with respect to the ring level both in the proximal and distal directions. On the proximal ring, a 5-whole-cube and a 1-whole-cube were used on the top side of the ring, and a 2-whole-cube was used on the bottom side of the ring. On the distal ring, a 4-whole-cube and a 1-whole-cube were used on the top side, and a 4-whole-cube was used on the bottom side (Figure 3). This resulted in a distance of 8.3 cm between the proximal and the distal half pin in the proximal ring-block and 9.8 cm in the distal ring-block. Fixation screws were tightened to 10 Nm.

A mid-diaphyseal osteotomy of size of 3.5 mm performed. The distance between the osteotomy and the inner rings measured 6 cm on either side in the wire frame and 10 cm in the half-pin frame.

**Figure 4:**
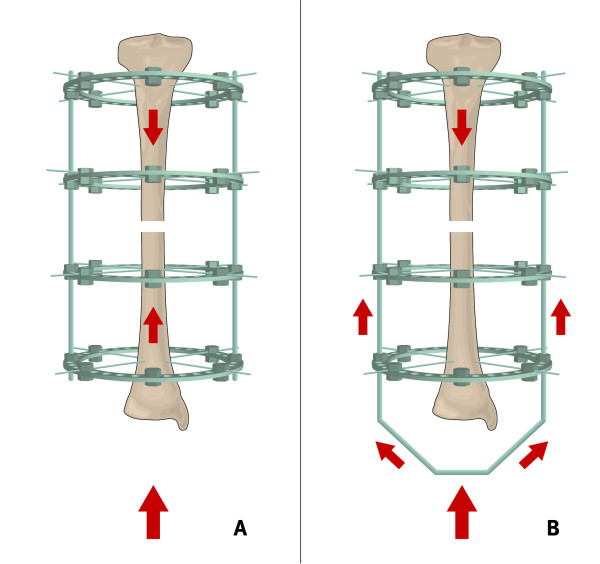
**Schematic drawing of the load transmission under direct (A) and indirect (B) loading**.

Two different loading configurations were analyzed (Figure 4): the loading of the bone with both ends fixed to the test machine, which is denoted as direct loading, and the loading of the bone with only the proximal bone end fixed to the test machine, which is denoted as indirect loading of the osteotomy gap. For the experimental direct loading set-up, both bone ends were mounted to the fixation plates. For the indirect loading set-up, the weight-bearing platform was simulated by distal extensions of the four connecting rods at the distal end of the frame, which was fixed to the base plate of the test machine. In this manner, the distal articular joint line of the composite bone did not contact the base plate of the test machine.

The composite tibiae were rigidly attached to a universal test machine (UTS^®^) with custom-made mountings (Figure 2). The parallel fixation plates allowed uniform axial loading along the mechanical axis of the tibiae. Continuous axial loading and unloading was applied to the bone at a velocity of 5 mm/min. The test machine was linked to a multichannel measuring system (MGC-Plus with ML55, HBM^®^). Inductive standard displacement transducers (WA T, HBM^®^) were used to measure the interfragmentary motion at the site of the defect, the relative motion of the bone fragments to the rings and the relative motion between the rings. There were three transducers at the site of the defect, two measuring the relative movements and three measuring the movements between the rings (Figure 3). For the measurement of the compressive loads in the osteotomy gap under direct and indirect loading, a loading cell (FGP Sensors^®^) was placed in the defect zone (Figure 2).

Axial loads up to 700 N were applied. Although most patients do not fully bear weight after initial frame application due to pain, a maximum weight load might possibly be experienced due to accidental slips or in patients that lack pain perception due to polyneuropathy.

To document the reproducibility, each test was repeated ten times with new wires and pins for each test. The load/displacement curves obtained from the averaged data for each ring configuration were analyzed with respect to slope and interfragmentary movement. The slope of the regression line of these average data points is defined as the frame's stiffness [12]. In this study, axial stiffness was determined using a regression between 100 and 200 N of axial loading because it reflected an intermittent linear regression in the load/displacement curves for each frame configuration. Additionally, the amount of axial load needed to cause one millimeter of interfragmentary compression in the osteotomy gap was determined, following the study of Khurana et al. [12], as it represents an important displacement range for beneficial axial micro-movements [21].

Data acquisition was performed using the VEE Pro software version 7 (Agilent Technologies^®^). The data were analyzed using an analysis of variance (ANOVA), and the Student's t test was used to compare correspomding compressive loads and stiffness values. Statistical significance was considered at p < 0.05, and all statistical analyses were performed using Microsoft Excel^®^ and a commercial statistical software package (Graph Pad Prism^®^, version 5.0).

## Results

### Compressive loads at the osteotomy site

**Figure 5:**
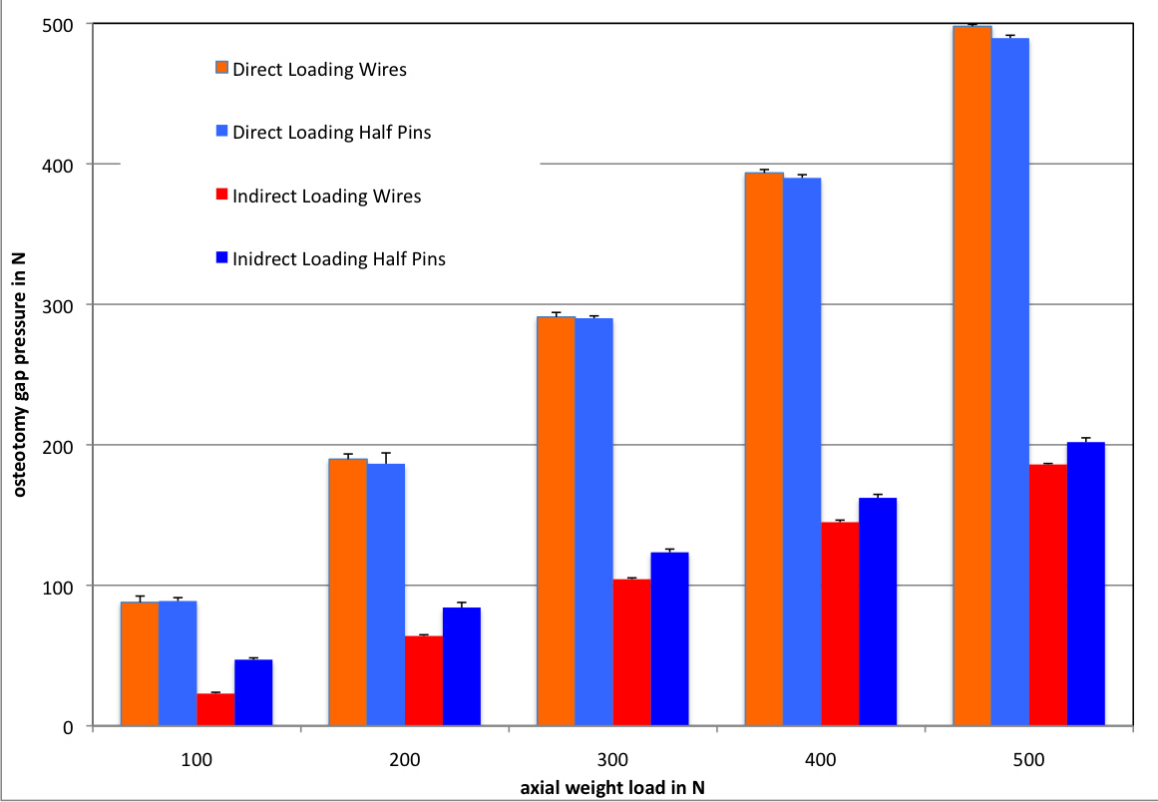
**Mean osteotomy gap pressures with standard deviations; x-axis: axial weight load (N); y-axis: osteotomy gap pressure (N)**.

Axial loading resulted in compression of the fracture ends. In direct loading, where both bone fragments were fixed to the test machine, the fragments were simultaneously pushed towards the osteotomy gap. At an axial weight load of 200 N, which simulates partial weight bearing in the clinical situation, a mean compressive load in the osteotomy of 189.76 (+/- 3.73) N in the wire frame and 186.52 (+/- 7.72) N in the half pin frame was measured under direct loading. Under indirect loading of the frame, 200 N of axial loading resulted in a compressive load of 63.92 (+/- 0.87) N in the wire frame and 84.18 (+/- 3.62) N in the half pin frame. Altogether, an average of 95% (+/- 3%) of the applied load was transferred through the osteotomy in the wire and the half-pin configurations without a significant difference (p > 0.05). Indirect loading of the composite tibiae resulted in the pushing of only the proximal bone fragment distally into the defect site and against the distal fragment with the abutment of the transfixating wires or half-pins. This reduced the compressive loads in the osteotomy by 67% of the induced axial load in the wire frame and 57% in the half-pin configuration. The differences between wire and half-pin mountings were statistically significant (p < 0.05), with greater differences observed under smaller loads and an approximation of the pressure values under larger loads. Figure 5 shows the comparative compressive loads at the osteotomy site for the wire and half-pin mountings under direct and indirect loading.

### Interfragmentary movements and axial stiffness

The results from the displacement transducers for the relative movement of the bone showed that at direct weight loading, both bone fragments were pushed towards each other in the direction of the osteotomy. The proximal and the distal fragment covered the same distance in relation to a ring level, which is half of the osteotomy gap size. Upon contact of both bone ends in the osteotomy, there were no more relative movements of the fragments. Under indirect loading, only the proximal fragment moved distally, in the direction of the osteotomy and the distal fragment. The proximal segment covered the total defect distance of 3.5 mm. The interfragmentary movements under direct loading were thereby a summation of the movements of both the proximal and distal bone fragments. This led to higher interfragmentary movements under equal loads compared to indirect loading. For example, an axial weight load of 200 N under direct loading caused interfragmentary movements of 1.89 (+/- 0.12) mm in the half-pin frame and 2.72 (+/- 0.16) mm in the wire frame. The same axial weight load resulted in a movement of only 0.97 (+/- 0.09) mm in the half-pin frame and 1.41 (+/- 0.11) mm in the wire frame. The axial loads required to cause a 1-mm displacement were significantly smaller under direct loading than in the case of indirect loading (p < 0.05). Under direct loading, 1 mm of axial interfragmentary movement occurred at a mean of 70.73 (+/- 0.80) N in the wire frame and 106.34 (+/-2.78) N in the half-pin frame. Under indirect loading, where only the proximal bone fragment was pushed into the osteotomy gap, the axial loading force almost doubled: 131.19 (+/- 3.96) N in the wire frame and 205.94 (+/- 5.60) N in the half-pin frame. A similar effect was observed for the axial stiffness of the frames. The wire frame demonstrated 93% higher stiffness under indirect loading compared to direct loading (p < 0.05), whereas the half-pin frame showed a 90% increase in stiffness (p < 0.05). A comparison between the wire and half-pin configurations demonstrated that the half-pin frame had 25% greater stiffness than did the wire frame under both direct and indirect loading (p < 0.05). The mean stiffness values and the required forces to create one millimeter of displacement are listed in Tables 1 and 2, respectively.

**Table 1:** Mean axial load to induce one millimeter of axial movement.

	Direct Loading	Indirect Loading
Wires	70.73 (+/- 0.80)	131.19 (+/- 3.96)

Half Pins	106.34 (+/- 2.78)	205.94 (+/- 5.60)

All values are in Newtons, and the standard deviations are given in brackets. The increased forces between wires and half-pins as well as between direct and indirect loading were significantly different (p < 0.05).

**Table 2:** Axial stiffness.

	Direct Loading	Indirect Loading
Wires	77.01 (+/- 0.24)	149.28 (+/- 2.26)

Half Pins	102.80 (+/- 1.74)	195.04 (+/- 3.58)

All values are given in N/mm, and standard deviations are given in brackets. The greater axial stiffness of the half-pin frames was statistically significant (p < 0.05). Indirect loading also induced a significant increase in stiffness compared to direct loading both in the wire and the half-pin configurations.

**Figure 6:**
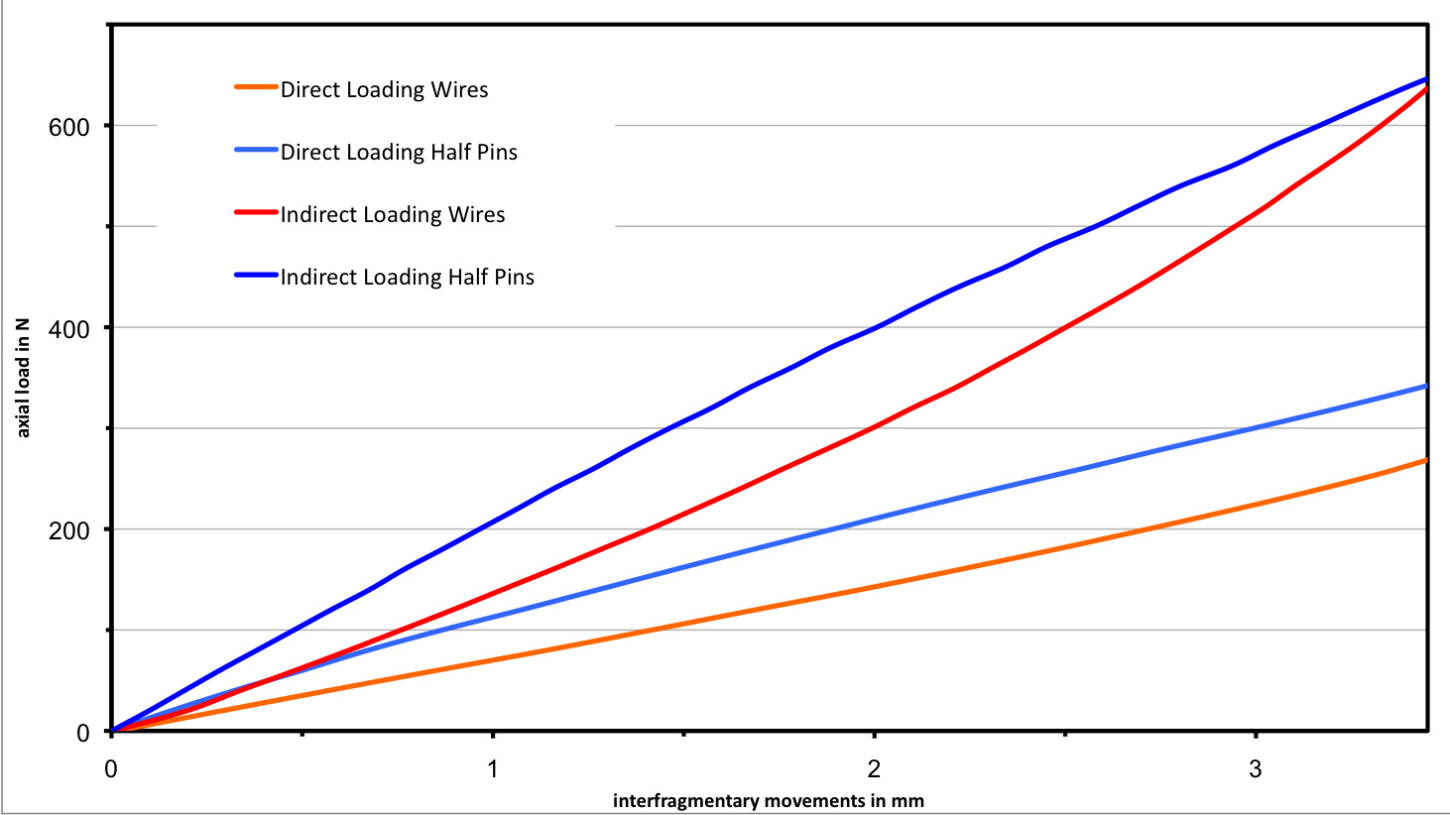
**Load-displacement curves for the different frame configurations; x-axis: interfragmentary movements (mm); y-axis: induced axial load (N)**.

The load displacement curves (Figure 6) for direct loading indicate an almost linear relationship between the induced load and interfragmentary movements. This linear relationship was demonstrated under indirect loading only for smaller loads below an approximate threshold of 250 N. With increased loading, both load-displacement curves showed a non-linear relationship. The wire frame demonstrated stiffening under increasing axial loading, whereas the load-displacement curve of the half pin frame leveled off with increasing load.

In the wire configuration, axial loading resulted in pure axial fracture site displacement both under direct and indirect loading. The proximal and distal bone fragments moved along the axis of the axial applied load, which led to a uniform gap closure.

**Figure 7:**
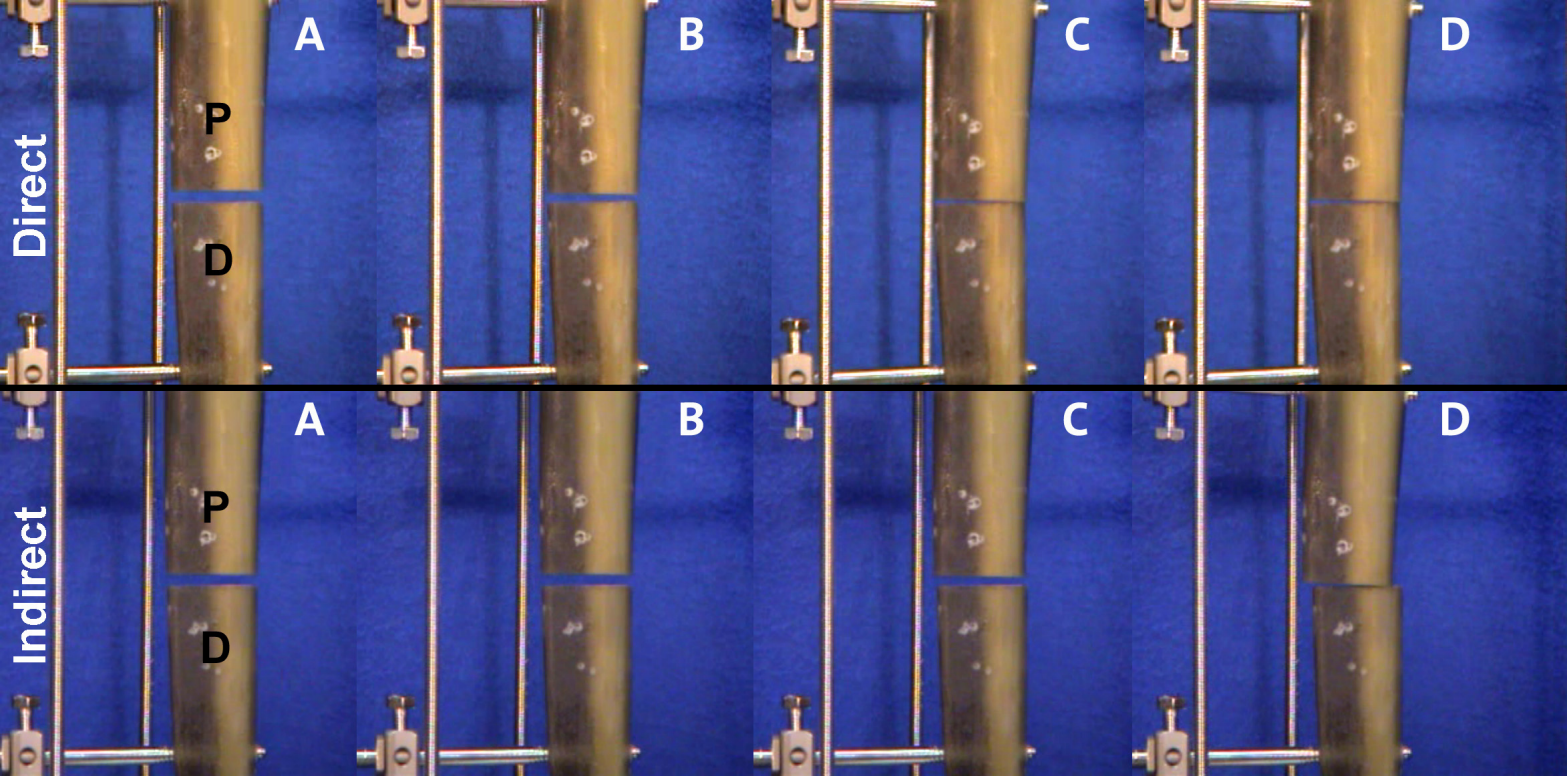
**Picture sequence demonstrating the translational displacement in the half-pin mounting at 0 N (A), 100 N (B), 300 N (C) and 600 N (D) under direct (top row) and indirect loading**. All measurement devices have been removed in this sequence.

In the half-pin configuration, axial loading produced translational movements, which resulted in angular displacement at the osteotomy (Figure 7). Under direct loading, both fragments simultaneously moved in the same direction such that the rear edges of the tibia were pressed against each other with no lateral displacement. Indirect loading in the half pin frames led to angular movement of only the proximal fragment, whereas the distal fragment remained in its initial position. This resulted in a secondary lateral shift of the proximal fragment relative to the distal fragment at the site of the osteotomy.

### Relative movements between rings

No instability of the connecting struts was detected. The maximum axial displacement between the rings under direct and indirect loading was 0.5 mm (SD = 0.06) in all configurations. There were no differences recorded by the displacement transducers between the various ring configurations (p > 0.05).

## Discussion

This study was designed to determine the previously overlooked effects of a weight-bearing platform in a four-ring, all-wire frame and a two-ring, all-half-pin frame. The problems faced by biomechanical studies of the Ilizarov external fixator also proved to be limitations of this study and are caused by the complexity and the infinite number of possible configurations. Any change in mounting parameters or osteotomy patterns directly influences the biomechanical conditions at the defect site [23,24]. The effects on frame stiffness of various mounting parameters (e.g., ring size, wire/half-pin diameter, number of wires/half-pins/rings) have been analyzed previously [6,12,23,25]. Therefore, the two frames were mounted as they are used in clinical practice with the wires and half-pins positioned with respect to the anatomic constraints of the tibia. As in other studies [5,26], only uni-directional axial loads were applied, although more complex loading conditions are present under weight bearing in clinical situations. Additionally, the influence of soft tissues, the stabilizing effect of an intact fibula and the natural stabilization due to bony healing cannot be studied with this composite bone model. These limitations must be kept in mind when interpreting the results.

An external fixator is mainly responsible for the load transfer through a fractured bone: the fixator functions as a mechanical bridge between the fractured bone ends that allows interfragmentary movements, which depend on the stiffness of the fixator [27]. The normal load path when bearing weight is through the bone, through the wires or pins of the external fixator, through the rings and connecting struts of the fixator at the level of the fracture and back through the wires or pins and into the bone [28]. Bony contact in combination with compression at the fracture site augments the frame's stability, which results in load sharing between the frame and the bone and the ability to bear weight [7,29]. With full contact of the bone ends in a plane osteotomy, all axial forces are transmitted through the osteotomy instead of the fixator [30,31]. Accordingly, in vivo measurements have recently shown that the maximum axial load in a fixator at the beginning of the healing process without bony contact is the body weight [27]. The results of the present study demonstrated that these biomechanical principles are changed by a weight-bearing platform; in a defect situation, the axial weight load is transferred only through the proximal wires or half-pins into the proximal bone fragment. The compressive forces in the osteotomy are a result of the axial compression of only the proximal bone fragment and the stiffness of the counter bearing, which consists of the distal wires or pins. For the two frame mountings that were analyzed in this study, only 33-43% of the applied load was transferred through the osteotomy under indirect loading.

In addition to the compressive loading forces, Claes et al. demonstrated that the interfragmentary movements, rather than the load at the fracture site, are important for the healing process [32]. Axial micro-movements have been shown to be beneficial to bone healing, although the precise threshold at which they become adverse has not been defined [33]. In animal studies, axial movements of up to 1 mm were associated with faster healing rates [21]. However, excessive axial or off-site movements that result in shear are detrimental to bone healing [20]. Therefore, an effective frame mounting must discourage translational and angular motions while still allowing some dynamic axial movements. The extent of the movements can be controlled by stiffening the frame. For the traditional all-wire frames, many biomechanical parameters have been defined that affect stiffness [6,7,23,24,34]. It has been shown that all-wire frames with a two-level fixation of the bone segment are highly resistant to angular displacements of bone fragments; the frame limits interfragmentary shear and bending at the fracture/osteotomy site [7,10,15,35]. Half-pins are used with Ilizarov frames because they simplify application, induce higher rigidity in the frame and reduce soft tissue complications [5,24]. The "Rancho technique" enables the use of only one ring per bone segment when using at least three half-pins. Although it has been argued that half-pins provide axial micro-motions similar to wires [12], many studies have indicated that axial compression is coupled with translational and angular motion in half-pin mountings. Due to the asymmetric, unilateral fixation, half-pins function as cantilever beams that result in translational movements and angular displacement at the site of the osteotomy [15,36]. Yang et al. reported that a Ilizarov hybrid fixator with one wire and one screw on each ring behaved more like a unilateral fixator than a circular fixator [37]. In vivo measurements of tibial osteotomies treated with ring fixators that consisted of wires and half-pins showed that shear movements generally exceeded axial compression [38].

The results of the current study are consistent with these results. Although higher axial stiffness was achieved with the less bulky frame configuration, the uni-directional axial loading led to angular displacements and, therefore, shear at the fracture site. While the all-wire frame demonstrated a plane osteotomy gap closure with no angular displacements under both direct and indirect loading, the axial loading of the half pin frames led to displacement at the osteotomy site. The cantilever effect was more pronounced for indirect loading. Together with angular displacement, translational displacement occurred with respect to the distal (non-moving) bone fragment, which resulted in greater shear forces at the osteotomy.

Previous authors have reported a non-linear relationship of the load-displacement for the Ilizarov frame [7,23,39]. This relationship was not obvious for direct loading in this study, but this might be due to the very small defect size, which led to only a small deflection of the wire or half-pin because of the small relative movements of the bone segments. Indirect loading led to a larger deflection of the wires and bending of the pins in the proximal fragment, which resulted in a non-linear relationship between the applied loads and the interfragmentary movements. This has been attributed to a self-stiffening effect of the wires, which are more resistant to deflection as loads increase [7,23]. This effect causes relatively larger compressive loads in the wire frame under indirect loading for larger axial loads; greater deflection of the distal wires strengthens the counter bearing against the proximal bone fragment. However, increased loads in the half-pin frame demonstrated a decreased stiffness; the load-displacement curve inclined slightly with increasing load. Greater bending of the half pins seems to decrease the fragment's stability.

Direct loading resulted in large amounts of interfragmentary movements under small weight loads because both fragments are pushed towards each other. At an axial load of only 20 kg, which corresponds clinically to partial weight bearing, we identified movements of approximately 2 mm in the half-pin frame and 2.7 mm in the all-wire frame. From in vivo measurements of patients treated with an Ilizarov frame, Duda et al. [40] demonstrated interfragmentary movements as large as 4 mm in the early treatment phase under a partial weight load of 20 kg. Conversely, the results for indirect loading demonstrated that the amount of movement is decreased by 50% in with respect to the applied load. However, this is accompanied by permanent higher mechanical stress on the proximal wires or pins, which may result in earlier material yielding and cause loosening and breakage of wires or pins. Increased bending of the pins also leads to higher mechanical stress at the pin-bone interface and may cause early pin loosening [15].

## Conclusions

Although the absolute magnitudes of the strain and interfragmentary movements that are detrimental to bone healing have not been precisely defined [1,5,33,34] and considering the limitations of this in vitro study, the aforementioned biomechanical effects may help in estimating the size and direction of interfragmentary movements and the mechanical stress on the frame. This is important in determining the weight bearing for patients in the early treatment phase, particularly for patients without bone apposition. The following conclusions can be drawn:

• A weight-bearing platform attached to an Ilizarov frame leads to an indirect loading at the site of the osteotomy.

• Lower compressive loads in the osteotomy are achieved with indirect loading at higher mechanical stress on the frame.

• Pure uni-directional axial loading leads to fracture site shear and angular displacements in the half-pin frames, although the pins induce higher rigidity to the frame.

• Indirect weight loading in the half-pin mounting results in larger angular and translational displacements.

## Competing interests

The authors declare that they have no competing interests.

No benefits in any form have been received or will be received from a commercial party related directly or indirectly to the subject of this article.

## Authors' contributions

JG and DS carried out the experiments and data analysis. BJ helped with the experimental set up and data analysis. MC participated in data analysis and helped to draft the mauscript. TAS participated in study design and coordination of the study. All authors read and approved the final manuscript.
